# Controlled Wrinkle Patterning on Thin Films to Improve Hydrophobicity

**DOI:** 10.1021/acs.langmuir.4c00743

**Published:** 2024-06-13

**Authors:** Margherita Aghito, Gabriel Hernandéz Rodríguez, Carlo Antonini, Anna Maria Coclite

**Affiliations:** † Institute of Solid State Physics, Graz University of Technology, Petersgasse 16, Graz A-8010, Austria; ‡ Department of Material Science, University of Milano-Bicocca, Via Roberto Cozzi 55, Milano 20125, Italy

## Abstract

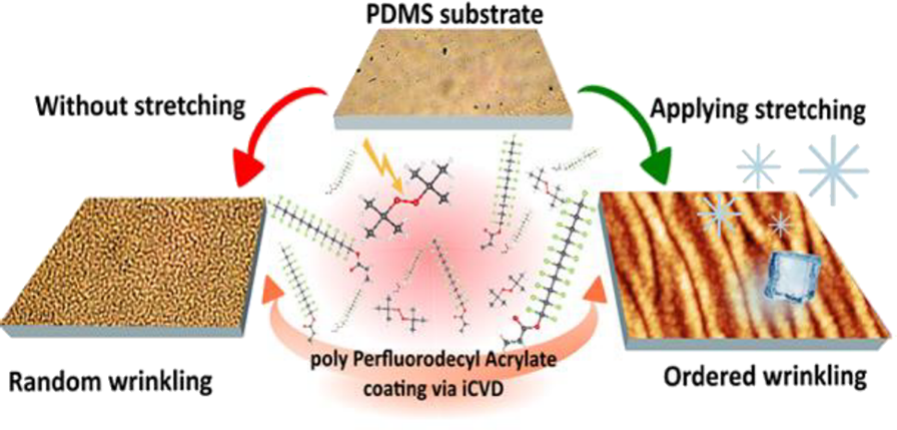

Controlling surface morphology is one of the main strategies used to tune surface hydrophobic and icephobic properties. Taking advantage of coating growth by initiated chemical vapor deposition, random and ordered wrinkles were induced on a thin film of polyperfluorodecyl acrylate (pPFDA) deposited on polydimethylsiloxane (PDMS) to simultaneously modify surface chemistry and morphology. A range of wrinkles of different wavelengths were studied, and how the wrinkle characteristics change with varying coating thickness. Ordered wrinkles enhanced hydrophobicity more when compared to random wrinkles, with a noticeable effect for coating thickness on the order of hundreds of nanometers. An insight into the mechanism of surface wrinkling and its effect on freezing delay is also provided, and promising results were found on ordered wrinkles, where a freezing delay was observed.

## Introduction

Over the last few decades, research has focused on studying wrinkling in both naturally occurring and artificially induced scenarios. Wrinkles can change the properties of the substrate on which they appear, for instance affecting the mechanical properties of a material or its conductivity.^1−3^ In general, wrinkles enhance the surface area, which can be beneficial to enhance transport phenomena in a variety of applications: e.g., micropatterning was found to affect the dissolution rate in drug release.^4^

Within the context of surface wetting, wrinkles with the right geometrical characteristics can be used to induce the so-called Cassie–Baxter state. When the Cassie–Baxter state is induced, the hydrophobicity of the surface is enhanced. A surface is considered hydrophobic when the water contact angle measured for a water droplet deposited on it is above 90°.^5^ Values above 150° for the water contact angle have been observed when inducing the Cassie–Baxter state, along with a hysteresis value ranging from 5 to 10°, thereby reaching the so-called superhydrophobic state.^6,7^ The Cassie–Baxter state can be described as a state in which a drop has low contact with the substrate, touching the top of surface asperities and with air pockets limiting the contact between the surface and droplet.^8^ This is why this state is also known as the *fakir state*. Although the Cassie–Baxter state is a metastable state, susceptible to transition to the Wenzel state, it can be obtained in special conditions, where the surface is patterned. An example of this is the induction of the Cassie–Baxter state through the presence of a micropattern distributed across the surface, such as in the case of micropillars.^9,10^

Wrinkles potentially represent a suitable configuration to obtain the Cassie–Baxter state^11^ and so to enhance hydrophobicity. Until now, it has been widely accepted that wrinkles can appear in substrates of varying magnitudes, depending on the characteristics of the substrate involved, as discussed by Rodríguez-Hernández.^10^ Wrinkles form when the top layer expands at a rate faster than that of the layer beneath it. In general, wrinkling can be understood as an out-of-plane surface bending that occurs due to instability under compression, induced by any parallel or perpendicular force above a certain stress threshold.^12^ With these ideas in mind, we want to focus on the random and controlled wrinkling induced by depositing a thin polymer film via initiated chemical vapor deposition (iCVD). iCVD is a versatile method to deposit polymer thin films because it has the advantage of being a completely dry process and it can be tuned to a high degree.^13^ Gleason et al.^14^ managed to induce a controlled wrinkling on a thin polymeric film of two combined monomers, ethylene glycol diacrylate and 2-hydroxyethyl methacrylate, deposited on polydimethylsiloxane (PDMS) via iCVD. Random wrinkles were also obtained by a previous work of our group exploiting iCVD to form poly-*N*-vinyl Caprolactam thin films on Eudragit surface.^15^ Wrinkling has been observed on Teflon layers deposited on various plastic substrates, resulting in a durable superhydrophobic state.^16^ This finding provides further evidence of the influence of wrinkles on the Cassie–Baxter state. Moreover, patterning induced under an electric field on piezopolymers proved the possibility of controlling the wetting properties.^17^

In the present work, we have studied how different wrinkle sizes can enhance hydrophobicity and freezing delay of a surface. For this, we induced wrinkling on a thin film of polyperfluorodecyl acrylate (pPFDA), deposited on PDMS via iCVD. pPFDA was selected as a coating due to its well-known hydrophobic properties, which arise from the abundance of fluorine atoms present in its molecular chains. In general, very high values of water contact angles (>130°) can be measured on such a material, depending on the degree of crystallinity of the polymer.^18^

## Experimental Section

For PDMS preparation, silicon elastomer base and curing agent (SYLGARD 184, Sigma-Aldrich) in a ratio 10:1 were used. A homogeneous mixture of the elastomer and curing agent was obtained in a beaker after magnetic stirring for 30 min. To eliminate most bubbles in the mixture, an ultrasonic bath was used for 15 min. A Petri dish was used as a mold for the substrates, obtaining PDMS samples 1 mm thick. The molds were placed in the desiccator under vacuum for 1 h, then in the oven at 70 °C for 1 h, and additionally at 90 °C for 1 hour. Samples with area 2 × 2 cm were cut from the molds. The PDMS samples were then ready for iCVD deposition, without further treatment. Differently, reference silicon samples that were used to monitor coating growth were cleaned in a mixture of ethanol/acetone 2:5, heated up at 185 °C, and then dried with Ar/CO_2_.

**Figure 1:**
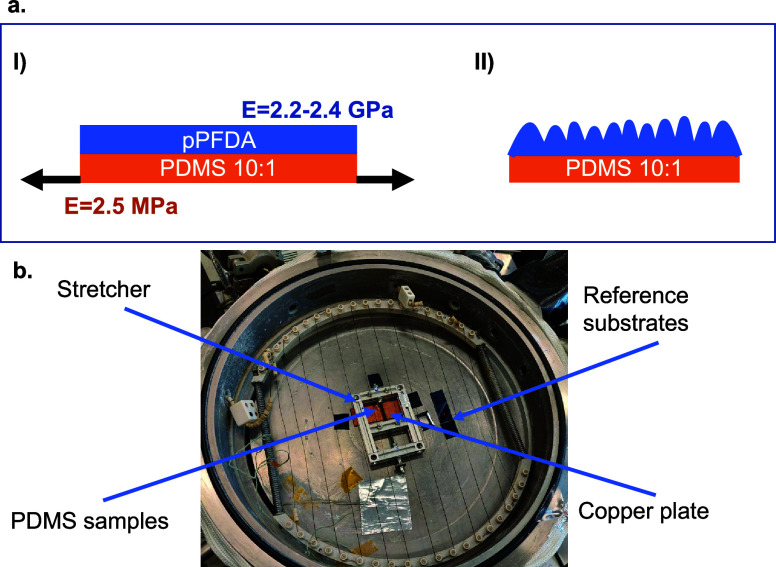
(a) Schematic representation of the stretching of the pPFDA-coated PDMS: (I) a 10% stretching is applied to the substrate during the coating process. The force is applied parallel to the substrate in a longitudinal direction. Both elastic moduli of polyperfluorodecyl acrylate (pPFDA) and polydimethylsiloxane (PDMS) are highlighted. (II) When the stretching is released, wrinkles appear. (b) The PDMS slice is placed in a self-built stretcher, put in contact with the cooling plate through a piece of copper. The stretcher is then surrounded by three silicon wafers, used as a reference to determine the thickness of the deposited coating via ellipsometry and to proceed to further characterization.

The synthesis of the coating was performed in a custom-built iCVD reactor.^19^ The monomer perfluorodecyl acrylate (PFDA, Aldrich 474487-25 ML) was heated to 85 °C and flown into the reactor at a rate of 0.200 ± 0.005 sccm. The initiator was kept at room temperature and entered the reactor with a flow rate of 1.0 ± 0.3 sccm. The working pressure was 400 mTorr. The hot wires were heated with a current of 1.15 A, reaching an average temperature of 180 °C, measured through a thermocouple. The reactor was heated to 60 °C, and the substrate holder was kept at 40 °C by a cooling system. A growth of 2.5 ± 0.1 nm/min was observed for pPFDA in these conditions. The PDMS samples were positioned in the stretcher and stretched up to 10% of their length (Figure 1a,b). After the deposition, the stretching was released, resulting in a sample appearance change from transparent to milky due to wrinkle formation. The coatings were grown up to thicknesses of 100, 150, 200, 300, and 600 nm. In addition, as a reference, some other PDMS samples were coated without applying any prior stretching, with thicknesses of 200 and 300 nm.

## Characterization Methods

Fourier-Transformed InfraRed spectroscopy (FTIR, Bruker IFS 66v/S) was performed on silicon pPFDA-coated samples to verify the fluorinated functional group retention and successful polymerization. Spectra were recorded in transmission mode in the range 500 to 4000 cm^–1^ with a resolution of 4 cm^–1^ (100 scans) and baseline-corrected (Figure S1, Supporting Information).

The coating thickness on the silicon references was then determined through spectroscopic ellipsometry (J.A. Woollam, M-2000 V). The samples were scanned at angles from 45° to 65° with a 10° increase, with an acquisition time of 2 s. Each coated silicon wafer was analyzed with a model consisting of the Si substrate, a 1.5 nm native oxide layer, and the polymer layer. For the latter, a Cauchy function was used.

An optical microscope (Olympus BX51 connected to an Olympus Camedia C-5060 camera) was used in transmission mode on the coated PDMS samples to observe wrinkles at different magnifications (5×, 50×, and 100×). Sample morphology and roughness were also analyzed by using an Atomic Force Microscope (AFM, Nanosurf Easyscan 2). A cantilever (Tap190Al-G, BudgetSensors, Bulgaria) working in tapping mode scanned areas of 25 μm × 25 μm and 50 μm × 50 μm (0.05 μm resolution) at a speed of 1 s/line. A 450 mV amplitude was employed, and the set point was set to 50%. The images were then elaborated through Gwyddion. Using the 2D-Fourier Transform analysis, it was possible to obtain the wrinkle wavelengths, whereas their height was measured by splitting each scanned image into five areas and averaging the peaks found. The water contact angle was measured by using a KSV CAM 200 goniometer. Droplets of various volumes (3, 4, 5, 7, and 10 μL) were deposited on the samples, and a camera was used to record drop profiles and extract contact angles. Advancing and receding angles were measured with a sessile drop test and used to calculate the hysteresis values.

The second part of the characterization included frost formation observations and droplet freezing tests. To observe the substrate behavior at low temperatures, a Linkam stage (model T95-PE) was used, coupled first with an optical microscope and then with an IR camera. The samples were first observed via an optical microscope at −45, −60, and −80 °C, reaching the desired temperature at three different cooling rates, namely, 15, 20, and 25 °C/min. Each time, the temperature was increased cyclically to room temperature. The aim of these preliminary tests was to determine whether visible changes would be observed on the wrinkle pattern.

Subsequently, the Linkam Stage was coupled to an IR Camera (Optris PI, 80 Hz). Five droplets of 10 μL were deposited at the same time on each substrate. The substrate was then cooled to −25 °C at 30 °C/min to observe the freezing delay, at 30% relative humidity. The freezing delay was calculated from the instant in which −25 °C is reached until the droplet starts freezing. The freezing delays measured for each droplet were averaged, and standard deviations were calculated. Lastly, the coating robustness was tested qualitatively by scratching frozen droplets from the substrate surface. After the samples were left with some droplets of random sizes in the freezer at −18 °C for one night, they were scratched away using plastic tweezers. To quantify the coating abrasion, a profilometer (KLA Tencor D-500) was used on the areas from which the droplets were scratched from.

## Results and Discussion

**Figure 2:**
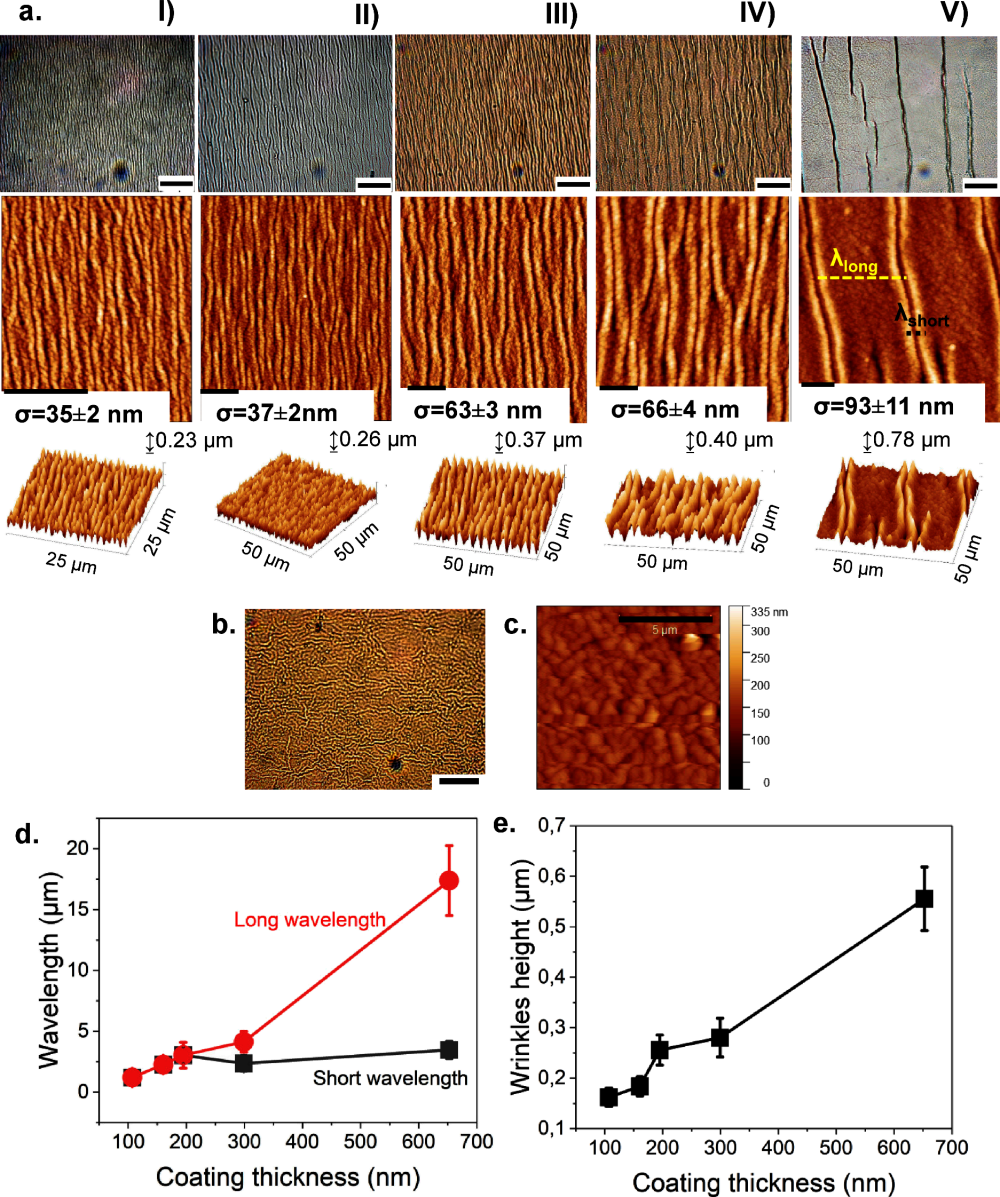
(a) Optical microscopy images (first row) and the AFM scans (second row) resulting from the five different coated surfaces are here shown: (I) 100 nm, (II) 150 nm, (III) 200 nm, (IV) 300 nm, and (V) 600 nm. The scale bar for the optical microscope images is 20 μm, while for the AFM images, it is 10 μm. In the third row, the 3D scan is reported, showing the 3D shape of wrinkles and the different distributions on the surface due to the change in thickness. On the right angle, the highest value for the height of wrinkles is reported. (b) Optical microscopy image of random wrinkles on a 200 nm coated PDMS with pPFDA. The scale bar is 20 μm. (c) AFM micrographs of the same surface, the scale bar is 5 μm. (d) Wrinkle wavelength versus coating thickness; two different wavelengths were identified: a long wavelength, λ long, and a short wavelength, λ short. The long wavelength shows the strongest influence from the increase of thickness, while the short wavelength has mostly a constant behavior. Both wavelengths are in the range of μm. e. This plot shows the height of wrinkles versus coating thickness: the increase is again linear. The plotted heights were obtained by averaging the wrinkle heights measured by AFM.

The first goal of our study was to understand how the coating thickness affects the wrinkle shape. Specifically, we studied the effect of coating thickness on the wrinkle wavelength and height, as it is known from previous studies that these parameters are directly proportional to the coating thickness.^10^ In all samples, wrinkles were oriented perpendicular to the direction of the applied stretching, indicating a consistent pattern (Figure 2a). Samples I and II exhibited a comparable shape, likely due to their relatively close wrinkle heights. For samples III–V, the distance between wrinkles increased with thickness. This also led to an increase of the wavelengths, as will be discussed later. In Figure 2a*,* the second row shows the AFM scans for each sample. Wrinkles in samples IV and V presented a double peak, so two wavelengths could be obtained through the 2D-FFT analysis. Differently, for samples I–III, only a single wavelength was found. Random wrinkles were observed on PDMS samples that were not prestretched. The shape of random wrinkles differs considerably from the ordered wrinkles on stretched PDMS, as visible in Figure 2b,c. The wavelength is rather small, ranging between 1.5 up to 2 μm, and their height closely corresponds to the thickness of the deposited coating. However, the measured roughness is lower than that found on samples with ordered wrinkles with the same coating thickness. The overall roughness on the surface of the 200 nm sample with random wrinkles measures 55 ± 5 nm.

As shown in the plot in Figure 2d, the distance between the double peaks is described by a short wavelength (λ_short_), while the distance between two contiguous double peaks is a long wavelength (λ_long_). The short wavelength appeared to be weakly affected by the increase in thickness, as it remained approximately constant. On the contrary, the long wavelength was highly enhanced by the thickness, reaching a value of almost 16 μm on the 600 nm coated sample.

Between the wrinkles, the surface appears uneven, thus leading to a difference in roughness over the surface. Nevertheless, the overall roughness increased with the coating thickness, as already reported in the literature.^20,21^ Defects and porosity are introduced into a pPFDA coating during synthesis via iCVD,^22,23^ as the coating becomes thicker. This leads to an increase of surface roughness.^24^

In Figure 2e, the wrinkle height is shown as a function of the coating thickness. It appears that increasing the thickness also leads to an increase in the height of wrinkles, consequently influencing the pattern shape. This was also confirmed by the 3D scan of the surfaces, shown in Figure 2a*,* third line. Note that wrinkle height ranges around the coating thickness.

### Hydrophobicity Characterization

To assess hydrophobicity and how this is affected by wrinkles, static water contact angle was measured.^25,26^ The reference value of water contact angle for uncoated PDMS is 120 ± 2°, measured at a room temperature of 25 °C. Therefore, PDMS can already be considered hydrophobic.^27^ The water contact angle measured on pPFDA deposited over a silicon wafer was 133 ± 2°. The fluorinated polymer is highly hydrophobic and highly crystalline when deposited on bare silicon.^26,27^

**Figure 3:**
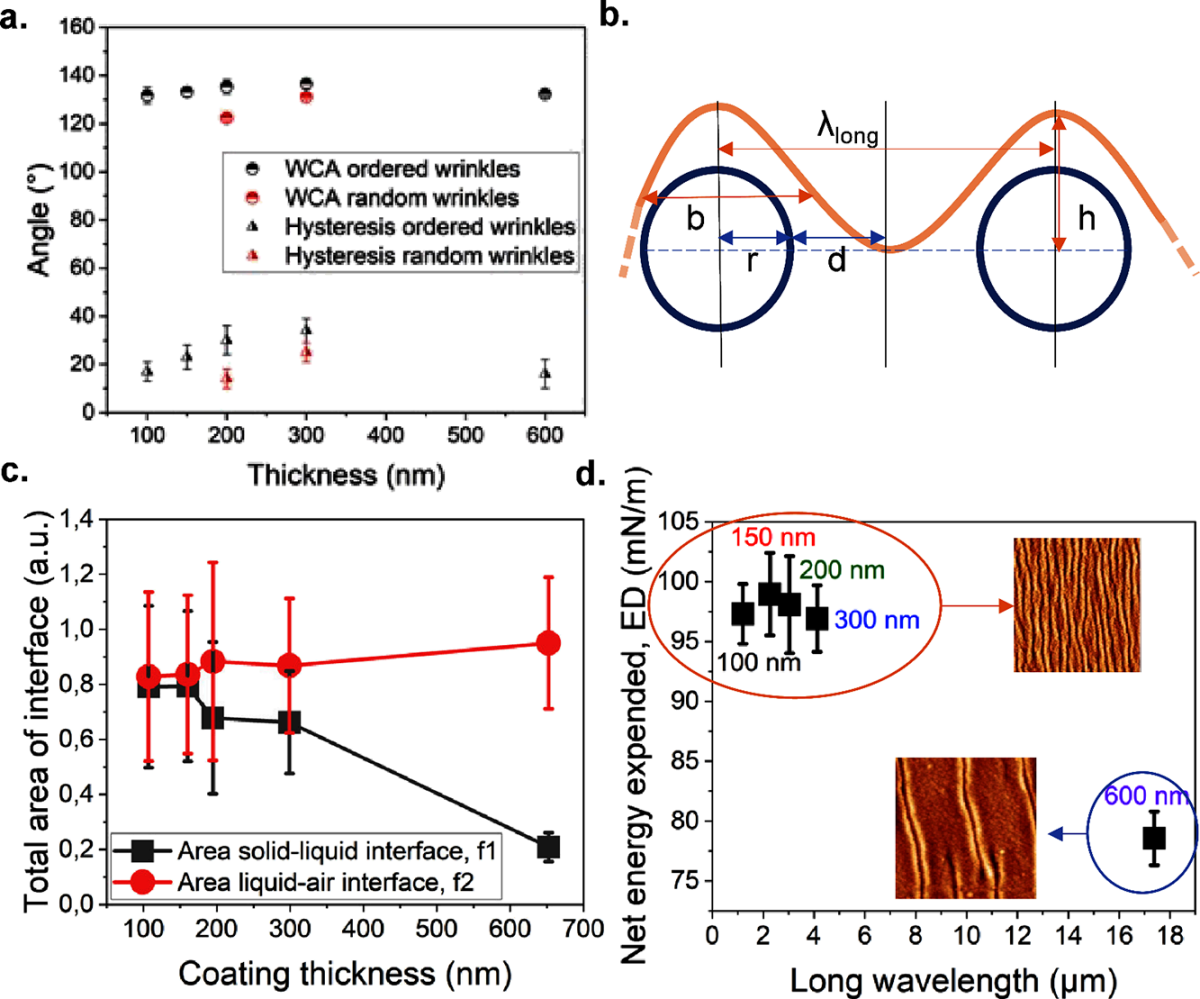
(a) Water contact angles were measured on several samples with ordered and random wrinkles. The error bars are indicated for each data point in the plot, but sometimes, they are hidden by the symbols. An enhancement in the WCA could be seen already in the 100 nm coated sample with ordered wrinkles, in comparison to the random wrinkles. (b) Schematic representation of how the Cassie–Baxter theory, originally developed to study systems based on fibers, was adapted to a wrinkled system to calculate the areas of interface and the energy spent to originate a Cassie–Baxter state. The parameters used to describe the fibers (radius, *r*, and middle distance between fibers, *d*) were related to the parameters of wrinkling (long wavelength, λ_long_, and width, *b*). (c) In this plot, the total area values of solid–liquid, *f*_1_, and solid–air, *f*_2_, interfaces are represented. (d) The net energy (ED) expended in forming unit geometrical areas of interface is plotted with the long wavelength: the shape of wrinkles determines a clustering of the values.

Figure 3a shows the water contact angles measured on the ordered and random wrinkles: both the thickness and patterning affected the water contact angle.^28−31^ The ordered wrinkles resulted in higher static contact angles compared to random wrinkles but also slightly higher contact angle hysteresis, probably due to a pinning effect derived from the peculiar shape of surfaces. For the sample with a 100 nm coating and ordered wrinkles, the water contact angle was higher than that of the 300 nm coating with a random pattern. As the coating thickness increased from 100 to 300 nm, the water contact angle increased to 140°. However, the impact of the ordered pattern diminished when the thickness exceeded 300 nm. The 600 nm coated sample showed a comparable value to thinner coatings, indicating that the wavelength of wrinkles was too long to interact effectively with the water droplet. Higher water contact angle values were measured on hierarchical wrinkles on Teflon by Scarratt et al.^16^

The Cassie–Baxter model is useful to calculate the nondimensional area of solid–liquid and liquid–air interfaces, *f*_1_ and *f*_2_, in the system and understand if the Cassie–Baxter state can be induced by the morphology of the wrinkles. From the total areas of the interface, it is possible to estimate the surface energy required to generate the Cassie–Baxter state.^6^ The Cassie–Baxter theory was developed for fabrics, so the equations used to study the system refer to fabric parameters. In this work, the model was adapted to wrinkles as shown in detail in the Supporting Information, Section 2. In Figure 3b, we show the overlap of wrinkles and fibers to define the corresponding approximations. The wrinkles on the samples were characterized by two different wavelengths: a short one and long one. In the model, only the long wavelength was considered, assuming that only droplets of relatively small dimensions could sit in a Cassie–Baxter state over the double peak of a wrinkle. In the graph shown in Figure 3c*,* the total interface areas are shown with the thickness of the coating. The solid–liquid interface area, *f*_1_, decreases with increasing coating thickness. This area represents the area of interaction between the wrinkles and droplet. At higher thicknesses, the distance between wrinkles increases and therefore the contact between the liquid and the wrinkles decreases, leading to the presence of air pockets (Cassie–Baxter state) between two consecutive wrinkles and the droplet. Nevertheless, this effect depends also on the height of the features, a parameter not included in the model. However, for the sample with thickness <300 nm, the height-to-wavelength ratio is high enough to ensure that the droplets are not sinking into the area between wrinkles. In the case of the 600 nm sample, this is no more verified, since the droplets were observed mainly in the flat area between wrinkles. This scenario is not described by the model, which only considers the area of interaction between wrinkles and droplet. Since the wrinkles are largely apart, the area of interaction with the droplet is small, which should lead to a decrease in the WCA. In the measurements for the 600 nm thick sample, though, the droplet sinks between wrinkles, and the Cassie–Baxter theory is no more verified. Only a slight increase in the total liquid–air area of the interface, *f*_2_, is observed with the coating thickness.

Figure 3d shows the net expended energy, ED, required to generate or consume interface areas as a function of the long wavelength. What influences most the entity of ED is the wrinkle shape. Indeed, when the wrinkles showed higher wavelengths (i.e., for the 600 nm thick sample), less energy was needed to generate the interface area. This agrees with the plot of *f*_1_ and *f*_2_: when the wavelength was higher, the solid–liquid area of the interface, *f*_1_, was lower, and the liquid–air interface area, *f*_2_, was only slightly increasing. A lower area of the solid–liquid interface was found when there were fewer wrinkles on a specific area (e.g., greater wavelengths). Therefore, the net energy expended to form the interface area was lower.

**Figure 4:**
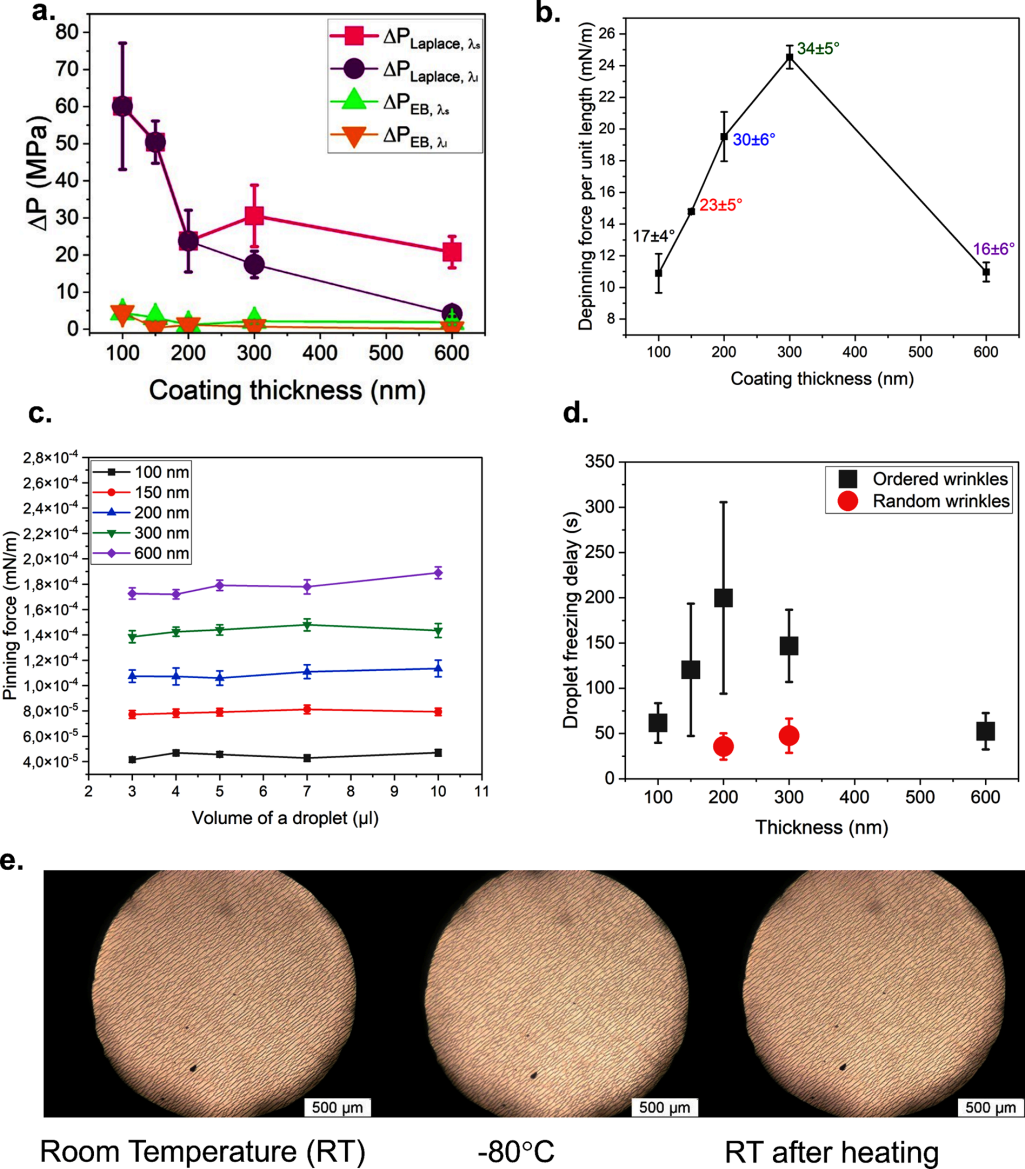
(a) Values for the Laplace and energy barrier pressure were calculated for each sample, considering both wavelengths where possible. Here, they were plotted with the thickness of the coating. (b) The depinning force per unit length could be calculated for each sample; it is related to the hysteresis values, which are reported for each sample. Going further with the thickness of the coating results in a decrease of the depinning force, and the 600 nm coated sample shows characteristics similar to those of the thinner coated samples. This is because different roughnesses are observed in different areas of the sample. (c) Plot of the pinning force over the volume of the droplet used during the tests, respectively, 3, 4, 5, 7, and 10 μL. An increase in the calculated pinning force is observed with the thickness, finding the 600 nm coated sample with the highest value. (d) Plot of delay in freezing measured for a droplet at the enhancement of thickness. Results are shown for ordered (black) and random (red) wrinkles. Multiple droplets on the surface of 10 μL, *T* = −30 °C, 3 cycles were run and averaged. The large error bars are due to inhomogeneities on the PDMS thickness derived from its preparation. (e) Images taken during the cooling to −80 °C and the heating up to RT at a cooling rate of 15 °C/min. 50× magnification, optical microscope, Olympus BX51.

Two forces act on the droplet to keep it suspended between two wrinkles: these are the Laplace pressure, which pushes the droplet down toward the surface of the sample, and opposite pressure related to the energy barrier to overcome when the Cassie–Baxter state transits to the Wenzel state. Figure 4a shows the Laplace and energy barrier pressures calculated from the model as a function of the coating thickness. First, both pressures are of the same order of magnitude, meaning that the pressure exerted by the surface could efficiently contrast liquid penetration. A decrease in the Laplace pressure is observed when the coating thickness is increased, because of higher wavelengths. The pressure is higher in shorter wavelengths. Instead in the 600 nm sample, the energy barrier pressure equals Laplace pressure. Also, when larger droplets were tested, a larger depth of penetration has been calculated (Figure S2, Supporting Information). Since the wavelength is strictly connected to the coating thickness,^10,16^ the droplet penetration increases with coating thickness. Moreover, the activation energy related to the transition Cassie–Baxter to the wet state, for both wavelengths, was calculated and found to be correlated to the thickness. However, while the one related to short wavelength is directly proportional to it, the activation energy related to long wavelength is indirectly proportional to the thickness, as shown in Section 3, Supporting Information in detail.

Finally, we evaluated the pinning force and force needed to remove a droplet from the surface; the description is brought on in detail in the Supporting Information, Section 3. The pinning force for the samples with ordered wrinkles is shown in Figure 4c, versus the volume of the droplets. The volume did not affect the entity of the pinning force, while when the thickness increases, the pinning force tends to increase as well. The depinning force (Figure 4b) needed to remove the droplet from the surface depends on the hysteresis. Quantifying this force gave information directly on the stickiness to the surface without considering the dimensions of wrinkling. The calculated depinning force was quite higher than the pinning force, as calculated. Although the 200 and 300 nm coated samples were the best performing with regard to hydrophobicity, they showed a greater hysteresis associated with a higher force needed to remove the droplet from the surface. The 600 nm coated sample proved once more to behave similarly to the thinnest samples. An explanation was found by measuring the roughness of these surfaces. As previously mentioned, the overall roughness increased with the thickness. On the 100 nm coated sample, the roughness was found to be 35 ± 2 and 37 ± 2 nm on the 150 nm coated sample. On the 600 nm coating, the overall roughness was 93 ± 11 nm. The high standard deviation derives from the inhomogeneity of the roughness over the surface. The measured roughness in the region between wrinkles was 42 ± 9 nm, and the coating appeared smoother already at first glance during the microscope analysis. This value is close to those found for the 100 and 150 nm coated samples. Differently, for the 200 and 300 nm coated samples, roughness of 63 ± 3 and 66 ± 4 nm were respectively measured. Moreover, the lower values of hysteresis found on the 600 nm coated sample suggested that the droplet has a favorite position, which is between two wrinkles on the smoother surface.

It is noteworthy to mention that the stickiness of these surfaces could also be characterized by measuring the roll-off-angle upon tilting the samples. For the case of surfaces with ordered wrinkles, it was noticed that the direction of tilting, either parallel or perpendicular to the wrinkle direction, caused differences in the roll-off-angle determination. Preliminary results on this are shown in Figure S4, Supporting Information.

### Analysis of the Behavior toward Freezing and Icephobicity

The behavior of the substrates in freezing conditions was investigated to understand if wrinkle structures change at low temperatures and if water droplet freezing is affected by wrinkles. The goal was to assess the substrates icephobicity potential, and specifically assessing the ice nucleation delay.^32^ First, we checked that wrinkles preserved their shape even after cooling down to −80 °C. In Figure 4e*,* the 600 nm thick coated sample is shown. No changes were observed in the wrinkle pattern, as the coating appeared unaffected by the cooling process. However, during the cooling, it was possible to observe the thermal contraction of PDMS.^33^ By heating up the substrates to room temperature, the original shape was recovered. A simple observation of the surface through an optical microscope did not reveal any wrinkle pattern modification. Thus, it can also be inferred that there was no change in height as the wavelength remained constant. We concluded that the coating is unaffected by low temperatures, and changes in the shape of the substrate did not alter it.

With respect to water drop freezing, nucleation started at the air–liquid interface^34^ (see the picture in Figure S5a, Supporting Information). The freezing front propagated toward the bottom of the droplet starting from the outer surface, and two phases could be distinguished. This was common among the pPFDA-coated samples: indeed, it was possible to remove the droplets from the surface of the substrates since the bottom part had a lower adhesion to the substrate surface. The experiment was repeated on a pPFDA-coated silicon sample, finding that the coating was damaged by the droplet (Figure S5, Supporting Information). Due to the coating stiffness (*E* = 2.2–2.4 GPa)^35^ and the rigidity of silicon, the droplet ripped the coating during the freezing process.

Figure 4d shows the effect of wrinkles on the freezing of droplets. Random wrinkles can delay freezing respectively of 36 ± 15 s for the 200 nm and 48 ± 19 s for the 300 nm coated samples. Although just the presence of a pattern helped delaying the freezing, having an ordered pattern affects it even more. Observing the results for ordered wrinkles, the delay was above the threshold reached with random wrinkles already with the 100 nm coated sample. The 200 nm coated samples with ordered wrinkles showed the highest value of 199 ± 105 s.

The damage on the coating due to droplet removal can be observed in Figure S8, Supporting Information. The experiment was done at room temperature, and the substrate was previously at −18 °C. The droplets had a volume of 10 μL. Bare PDMS and silicon wafer were tested as well. The force needed to remove ice from the silicon surface with tweezers severely damaged the sample: this was expected since silicon is hydrophilic^36^ and ice sticks to its surface. Instead, with bare PDMS ice could be more easily removed without damaging the substrate. The pPFDA coating significantly reduced ice adhesion: simple touching of the frozen droplets with tweezers was enough to promote ice detachment. Iced droplets left round transparent areas on every coated sample, meaning that damage occurred. The results from profilometry proved that a few nanometers of the coating were removed in those areas (Table S4, Supporting Information). The sample with the 300 nm coating was damaged the most, probably due to its higher hysteresis and pinning force.

## Conclusions

Our study focuses on iCVD and has been used for the enhancement of hydrophobic properties of PDMS by depositing via iCVD a fluorinated coating with a wrinkled pattern. The use of ordered wrinkles resulted in better performance than random wrinkling, and controlling the wavelength and height of wrinkles allowed for higher water contact angles at lower thicknesses of coating. A crucial finding in our study is the identification of a threshold value that enhances the hydrophobic properties of samples, thereby minimizing the required chemicals for the process. Moreover, iCVD proved to be a suitable instrument for highly tunable and controllable synthesis. A correlation between wrinkling parameters and thickness of the deposited layer was found, and it was also possible to calculate the energy involved in the system wrinkle-water droplet adapting the Cassie–Baxter theory. Through the analysis, it was proven that achieving thicknesses greater than 200 nm did not improve substrate properties, in terms of hydrophobicity and freezing delay. A decrease in the effect of wrinkling was observed when the coating thickness exceeded a certain value, as for the 600 nm coated sample, because the higher the film thickness, the more distant were the wrinkles, resulting in a faster loss of the Cassie–Baxter state. Wrinkles were stable at low temperatures, and freezing delay experiments showed an increase in the freezing time, with a maximum delay of 200 s observed for 300 nm thick coatings. Even though these are still preliminary results, they show that wrinkling holds promise for improvement also of the icephobic properties of PDMS.

## Supporting Information Available

FTIR analysis of the coating, the Cassie–Baxter theory adapted to wrinkles, surface characterization and energy calculations, and icing experiments (PDF)
