# Supplementary material for: Controlled Wrinkle Patterning on Thin Films to Improve Hydrophobicity
Source: Langmuir. 2024 Jun 13;40(25):13017–24. doi: 10.1021/acs.langmuir.4c00743 (PMC11210287; doi:10.1021/acs.langmuir.4c00743)
Supplement: Supplementary file 1 — la4c00743_si_001.pdf [file la4c00743_si_001.pdf]

Supporting Information

Controlled Wrinkle Patterning on Thin Films to improve Hydrophobicity

Margherita Aghito[1,2], Gabriel Hernandéz Rodríguez[1], Carlo Antonini*[2], Anna Maria Coclite*[1]

[1]Institute of Solid State Physics, Graz University of Technology, Petersgasse 16, A-8010 Graz

[2]Department of Material Science, University of Milano-Bicocca, Via Roberto Cozzi, 55, 20126 Milano

*Corresponding author at:

Institute of Solid State Physics, Graz University of Technology, e-mail address: a.coclite@tugraz.at (A. Coclite)

Department of Material Science, University of Milano- Bicocca, e-mail address: c.antonini@unimib.it (C. Antonini)

Table of Contents

Section 1- FTIR analysis of the coating S2

Section 2 – The Cassie-Baxter theory adapted to wrinkles S2

Section 3- Surface characterization and energy calculation S4

Section 4- Icing experiments S12

References S15

Section 1- FTIR analysis of the coating

The composition of the coating was investigated through FTIR. The resulting spectrum is shown in *Figure S*1. In the range between 1100 and 1300 cm^{-1} the peaks relative to -CF_2- stretching (1210, 1240 cm^{-1}) were clearly visible, also the -CF_2-CF_3 peak at 1150 cm^{-1} was detectable. At 1750 cm^{-1} there was the C=O characteristic peak, present in the acrylate. Around 1700cm^{-1} there was a small peak related to the vibration of -C=C-, which means that the polymerization was not complete. However, the intensity is low in comparison to the -CF- Therefore the degree of polymerization was satisfying for the properties of the coating. At higher wavenumbers, around 3000 cm^{-1}, also the =C-H peaks were spotted. While around the 1000 cm^{-1} area were present the peaks related to the vibration of Si-O-Si present on the surface of the silicon wafer (1050 cm^{-1}).

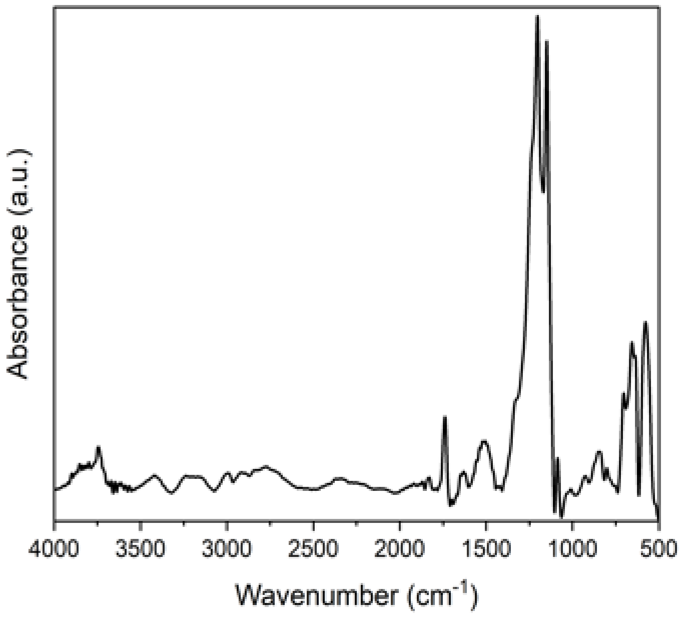

Figure S1- FTIR of a 100 nm pPFDA coating deposited on silicon wafer.

Section 2 – The Cassie-Baxter theory adapted to wrinkles

The Cassie Baxter theory needed to be adapted from fibers to wrinkles. Therefore some approximations needed to be done. A fiber can be described as a circular wire of indefinite length. Each fiber is then characterized by a certain radius, r. The center of a fiber is fixed in *o* and it is at a

defined distance from the next fiber[1]. The distance d between one fiber and the middle point to the next fiber is represented in *Figure 3-b*. The wrinkles on the samples were characterized by two different wavelengths, a short and a long one. In this study, only the long wavelength was considered, assuming that only droplets of relatively small dimensions could sit in a Cassie-Baxter state over the double peak of a wrinkle. When the wrinkled system is described, other parameters play a role: the wavelength, λ_{long}, the width, b and the height, h. Overlapping two wrinkles to two fibers next to each other, some resemblances could be found, in particular, the following approximation could be done:

$$r + d \approx \frac{\lambda_{long}}{2} \quad (1)$$

$$r \approx \frac{b}{2} \quad (2)$$

Starting from these relations, the Cassie-Baxter model for a wrinkle allows to define, f_1 and f_2, and the net energy, E_D:

$$f_1 = \frac{\pi r}{r+d}\left(1 - \frac{\theta_A}{180°}\right) \rightarrow f_1 \approx \frac{\pi b}{\lambda_{long}}(1 - \frac{\theta_A}{180°}) \quad (3)$$

$$f_2 = 1 - \frac{r\sin\theta_A}{r+d} \rightarrow \ f_2 \approx 1 - \frac{b\sin\theta_A}{\lambda_{long}} \quad (4)$$

$$E_D = f_1(\gamma_{sl} - \gamma_{sa}) + f_2\gamma_{la} \rightarrow \ E_D \approx f_1(\gamma_{ls} - \gamma_{sa}) + f_2\gamma_{la} = \frac{\pi b}{\lambda_{long}}\left(1 - \frac{\theta_A}{180°}\right)\gamma + (1 - \frac{b\sin\theta_A}{\lambda_{long}})\gamma_{la}(25°C) \quad (5)$$

Where θ_A is the advancing angle measured for each sample. The net energy considers the balance of energies involved in the stability of a liquid on a patterned surface. γ_{sa}, γ_{ls}, and γ_{la} are respectively the solid-air, liquid-solid, and liquid-air interface tensions. *Equation 3* represents the total area of the solid-liquid interface, f1. The radius r and the mid-distance between two fibers, d, were exchanged with the wavelength, λ_{long}, and the width, b, as for the other equations. The total area of the liquid-air interface is represented by f2 (*equation 4*) and, as for f1, it also depends on the advancing angle. When the interface areas are calculated, it is possible to understand if the Cassie-Baxter state can be induced

by the morphology of the surface. The last equation (*5*) regards the energy ED involved in the system considering all the new and old interface areas created when a droplet is deposited on a surface. When this happens, some energy must be spent because new areas of the interface are created, but since already existing interfaces are consumed, some of this expended energy is recovered. This can be seen in *equation 5* where $f_1 \gamma_{sa}$ is gained: some solid-air interfaces are consumed in favor of the new solid-liquid interface area, in fact, $f_1 \gamma_{ls}$energy is expended, and, due to the fact that some liquid-air interface area is consumed as well, also $f_2 \gamma_{la}$energy is expended. The interface tension values were derived from the Young-Duprée formula (*equation 6*), knowing the water static contact angles values:

$$\theta = \frac{\gamma_{sl}-\gamma_{sa}}{\gamma_{la}} \quad (6)$$

The values of $\gamma_{sl} - \gamma_{sa}$ were calculated as a difference, since the single values cannot be directly derived. γ_{la} is tabulated at RT (25°C), with a value of 71.99(±0.05) mN/m [2]. All the values used refer to a droplet of 5µl.

Section 3- Surface characterization and energy calculation

The system of a droplet laying on wrinkles was analyzed. The droplet wedged on wrinkles is applying pressure towards the surface of the sample, known as Laplace pressure [3]. This is due to its weight and it will eventually penetrate through the defects. The depth of penetration can be calculated through the Laplace pressure, as will be shown soon. The surface as well pushes upwards the droplet, preventing it to wet the internal part of the pattern and keeping stable the Cassie-Baxter state. The pressure exercised from the surface is related to the energy barrier to overcome when the Cassie-Baxter state transits to the Wenzel state, that is why it is called EB. If this latter pressure is not high enough, the Cassie-Baxter state would collapse into the Wenzel state. Also if the depth of penetration is greater the Cassie-Baxter state becomes harder to be maintained [4]. The presence of an energy barrier pressure prevents the droplets from wetting the surface of the sample. Considering the parameters of wrinkling, it was possible to estimate the pressure values in good approximation:

$$\delta \approx \frac{\lambda^2}{R} \tag{7}$$

$$\Delta P_{Lap} \approx \frac{\gamma_{la}}{\lambda_{long}} \tag{8}$$

$$\Delta P_{eb} \approx \frac{\pi \Delta E_{wenzel}}{2\sqrt{3}\lambda_{long}} \tag{9}$$

For the calculation of the depth of penetration, δ, both the wavelengths (λ) were taken into consideration for the 300 and 600 nm coated samples. The radius of the droplet R was calculated from the volume. Laplace pressure (ΔP_{Lap}) was determined from the liquid-air surface tension, γ_{la}, tabulated at RT, and it was calculated just for the long wavelength (λ_{long}), as well as the pressure related to the energy barrier (ΔP_{eb}). This last contribution can be calculated through the activation energy. In *Figure S2- I)* the depth of penetration for the short wavelength is shown over the radius of the droplet: when the droplet was bigger the depth decreased. In this situation, the droplet tended to sit better upon two wrinkles. Since the wavelength is strictly connected to the thickness of the coating [5,6], when there is an increase in the thickness also the penetration of the droplet is higher because there is more space between two wrinkles. This situation was common more specifically for smaller droplets.

In fact, when observing the results obtained for the long wavelength in *Figure S2- II)* the 600nm coated sample showed a deeper depth of penetration. The droplet of smaller volume, characterized by the lower radius, penetrated for more than half the average height of wrinkles. This put the system in the right condition to transit in a Wenzel state [7], even if as previously calculated it would have the lowest area of the solid-liquid interface. Where wrinkles are denser and closer, such as for the samples 100 and 150 nm coated, the depth of penetration is a few hundredths of µm. This means that the droplet had not the opportunity to go through the wrinkles. If the droplet is more able to penetrate that means that the Cassie-Baxter state can be hampered or deleted. Considering the long and the short wavelengths for the 300 and 600 nm coated samples, there was a great difference in penetration when the droplets sit on a double peak or between two wrinkles. While the droplets could not

penetrate much when sitting on a doubled peaked wrinkle, it was different when the space available between wrinkles increased.

When a droplet is upon wrinkles it has an internal pressure higher than the environment, a difference known as Laplace pressure (ΔPLap). The capillary forces sustain the meniscus upwards, preventing it to wet the internal part of the pattern and keeping stable the Cassie-Baxter state. The resistive pressure from the surface is related to the energy barrier (ΔPEB) to overcome when the Cassie-Baxter

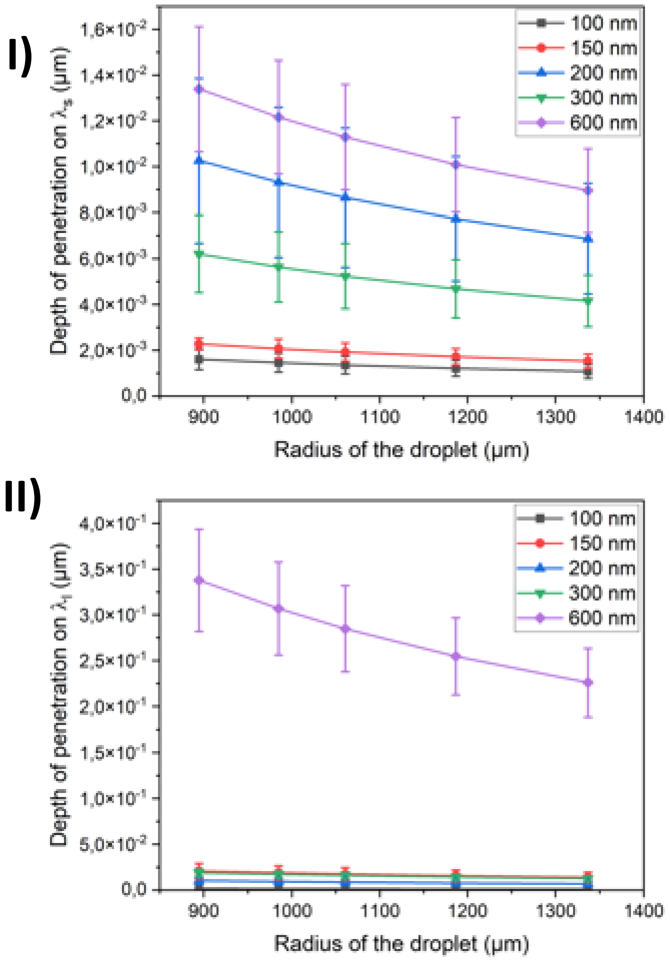

Figure S2- I) Depth of penetration calculated for the short wavelength, II) depth of penetration calculated for the long wavelength over the radius of the droplet, R

state transits to the Wenzel state. If this latter case, the pressure is not high enough, so the Cassie-Baxter state collapses into the Wenzel state. Also for higher depths of penetration, the Cassie-Baxter state becomes harder to be maintained.

As mentioned, the Cassie-Baxter state is a metastable state [1,8], while the wet state, or Wenzel state, is stable and at lower energy. If the energy needed for the transition is provided to the system by any external source, such as vibration, the Cassie-Baxter state will transit to the Wenzel state, and a certain quantity of energy will be released [3,8]. In principle, the Cassie-Baxter state could be stable for the entire existence of a system droplet over the surface, if the transition to the Wenzel state does not occur. The activation energy plays the main role, since if the system cannot overcome this barrier the transition will not happen. Therefore, the optimal conditions enhance the activation energy. Not only this but also if the Wenzel state is not stable enough the probability of a transition will decrease. The activation energy is influenced by the morphology of the surface and the surface tension of the droplet. It is proportional to wrinkling parameters, in this sense, it could be tuneable with them. Another quantity that is related to wrinkling parameters is the released enthalpy when the transition is observed. Together with the activation energy, it is important to the stability of the Cassie-Baxter state. In the following equations, the energy values are related to wrinkling parameters:

$$\Delta E_{Wenzel} \approx \frac{\pi h}{2\sqrt{3}\lambda}\gamma \qquad (10)$$

$$\Delta E_{C-W} \approx \frac{2\pi b h}{\lambda^2}\gamma \cos\theta \qquad (11)$$

The first equation represents the activation energy the system needs to reach the wet state. The values depend on the depth of penetration of a Cassie-Baxter droplet, previously calculated in *equation 7*. The liquid-vapor interface is assumed to not change as the drop sinks in the defects, only the amount of wetting of the inner side of wrinkles is changing.

The energy barrier is proportional to height and wavelength, both the long and the short. Moreover, the surface tension is calculated as the difference between the solid-liquid and liquid-air interface tensions, $\gamma = \gamma_{sl} - \gamma_{sa}$, derived from the Young-Dupré formula (*Table S1*). Both wavelengths were used for the calculation, where possible. Further implications allowed us to distinguish the wavelength when it was meant to be considered as long and short. It was found that, usually, the activation energy

found is massive enough that it could not be overcome with the thermal energy alone. When the Wenzel state was reached the droplet was strongly pinned in the defects and its shape changed due to a deformation imposed by the pattern. The transition was extremely quick, as explained by the Washburn law for the progression [9,10]. The mechanism has been described by Sbragaglia [11] as a zipping mechanism of the droplet during the transition from the Cassie-Baxter to the Wenzel state. A wet state results in higher values of hysteresis, characterized by low values of receding angle. In *equation 11* the enthalpy released after the transition is defined. The cosine of the static water contact angle and the width of wrinkles are influencing its value. Since the Wenzel state lays at slightly lower energy than the Cassie-Baxter state a negative energy contribution was found. The activation energy and enthalpy as calculated for the short wavelength grew with the thickness of the coating. The activation energy to overcome to reach the wet state resulted to be higher for bigger droplets. The situation was different when the long wavelength was considered for the calculation. We observed an inversion of the trend, and the activation energy increased when the thickness of the coating decreased. We can relate that a higher thickness did not help hampering the transition. This means it was easier to transit from the Cassie-Baxter to the Wenzel state in terms of kinetics [3]. We inferred this to the great enhancement of the wavelength. When the distance between two wrinkles was too large, droplets tended to roll in between. A more packed pattern of wrinkles affected the activation energy enhancing it. Also, the Wenzel state appeared to be more stable, presenting a lower enthalpy value. A correlation between activation energy and thickness of the coating was found. As mentioned, the Cassie-Baxter state is preferred to enhance hydrophobicity, thus tuning the activation energy related to the transition is one of the goals.

A correlation between activation energy for the transition between Cassie-Baxter to Wenzel state (ΔE_{Wenzel}) and coating thickness was found. As mentioned, the Cassie-Baxter state is preferred to enhance hydrophobicity, thus tuning the activation energy related to the transition could be used to tune the surface wettability. The activation energy for each sample was divided by the corresponding volume (3, 4, 5, 7, and 10 µl) of the tested droplets and plotted versus the logarithm of the coating

thickness. For the calculation relative to the long wavelength, $\lambda\, long$, the 150 nm coated sample was excluded because, as shown in Figure 2-d, its wavelength has the same value as the short wavelength.

$$\Delta E_{Wenzel,\ \lambda\, long} = \frac{10^{y_{int}}}{d^{0.8}} \quad (1)$$

Starting from the logarithmic equation, the activation energy was extrapolated through a linear fit (adjusted R-square of 0.95), and an inverse proportion was found with the thickness, as shown by *equation 1* (*Table S2, Supporting Information*). Knowing the volume of the droplet and the thickness of the coating the activation energy of the transition to the wet state can be predicted with good approximation. In *Figure 4- c* the linear fit is shown in pink. The same was done for the short wavelength, $\lambda\, short$, finding an adjusted R-square of 0.94 (Figure 4-d). In this second case, the outlier resulted in being the 100 nm coated sample, which was excluded from the fitting. A new relation was found to correlate the thickness of the coating to the activation energy when considering the short wavelength (*equation 2*), the plot is shown in *figure 4- d*. (*Table S3, Supporting Information*)

$$\Delta E_{Wenzel,\ \lambda\, short} = 10^{y_{int}} d^{0.6} \quad (2)$$

The samples which showed the highest activation energies for the transition between Cassie-Baxter to Wenzel state were the 100 nm coated sample for the long wavelength and the 600 nm coated

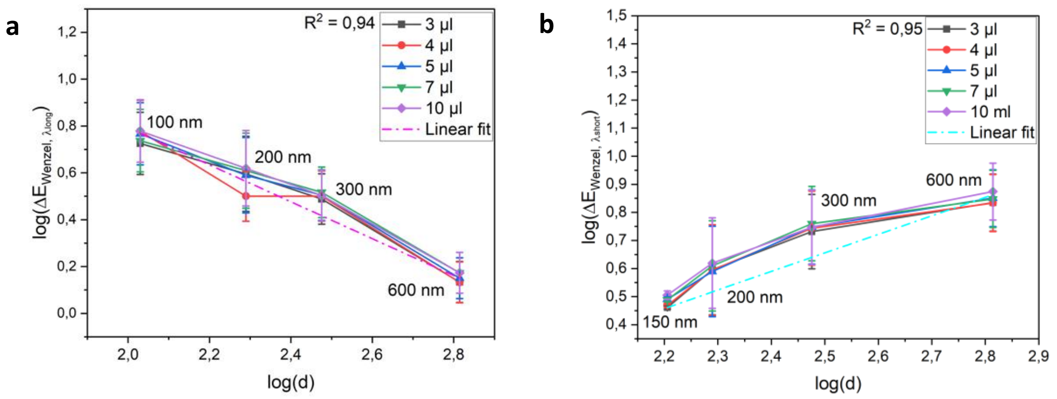

Figure S3- a. Plot of the logarithm of the activation energy for the long wavelength over the logarithm of the thickness. The 150 nm coated sample was excluded from the fitting since its wavelength could not be considered long. A linear fit is shown in pink. b. Plot of the logarithm of the activation energy for the short wavelength over the logarithm of the thickness. I this case it was the 100 nm coated sample to be excluded, since its wavelength could only be considered as long.

sample for the short one. This is in line with what experimentally observed, that on the 600 nm coating the drop sits between two consecutive wrinkles and therefore only the short wavelength could justify the formation of a Cassie-Baxter state.

Table S1- Data collected and calculated for each sample with ordered wrinkles.

**100nm**							
volume (µl)	±ε	WCA (°)	±ε	$(\gamma_{sl}-\gamma_{sv})/\gamma_{lv}$	±ε	$\gamma=\gamma_{sl}-\gamma_{sv}$ (mN/m)	±ε
3.15	0.03	127	4	0.60	0.02	43	1
4.2	0.4	133	3	0.68	0.02	49	1
5.2	0.2	131	3	0.66	0.02	48	1
6.9	0.1	128	6	0.62	0.03	45	2
10.0	0.1	133	5	0.68	0.03	49	2
**150nm**							
volume (µl)	±ε	WCA (°)	±ε	$(\gamma_{sl}-\gamma_{sv})/\gamma_{lv}$	±ε	$\gamma=\gamma_{sl}-\gamma_{sv}$ (mN/m)	±ε
3.2	0.1	129	2	0.630	0.01	45.6	0.6
4.0	0.1	130	2	0.65	0.01	46.5	0.8
5.2	0.1	133	3	0.68	0.01	48.8	0.9
7.0	0.1	132	2	0.68	0.01	48.6	0.8
10.3	0.2	135	5	0.70	0.02	50	2
**200nm**							
volume (µl)	±ε	WCA (°)	±ε	$(\gamma_{sl}-\gamma_{sv})/\gamma_{lv}$	±ε	$\gamma=\gamma_{sl}-\gamma_{sv}$ (mN/m)	±ε
3.1	0.04	136	2	0.72	0.01	52	1
4.0	0.1	136	6	0.71	0.03	51	2
5.1	0.1	135	4	0.71	0.02	51	2
6.9	0.1	138	3	0.74	0.02	53	1
10.1	0.1	139	5	0.76	0.03	54	2
**300nm**							

volume (µl)	±ε	WCA (°)	±ε	$(\gamma_{sl}-\gamma_{sv})/\gamma_{lv}$	±ε	$\gamma=\gamma_{sl}-\gamma_{sv}$ (mN/m)	±ε
3.1	0.2	134	3	0.69	0.02	50	1
4.1	0.1	136	1	0.71	0.01	51.4	0.4
5.2	0.1	136	2	0.72	0.01	52.0	0.7
7.0	0.1	138	3	0.74	0.02	53	1
10.2	0.2	136	4	0.72	0.02	52	2
**600nm**							
volume (µl)	±ε	WCA (°)	±ε	$(\gamma_{sl}-\gamma_{sv})/\gamma_{lv}$	±ε	$\gamma=\gamma_{sl}-\gamma_{sv}$ (mN/m)	±ε
3.1	0.1	131	2	0.65	0.01	47.0	0.8
4.0	0.1	131	1	0.65	0.01	46.8	0.4
5.2	0.1	133	2	0.68	0.01	48.7	0.6
7.2	0.3	132	3	0.67	0.02	48	1
10.2	0.2	136	2	0.71	0.01	51.4	0.8

The roll-off angle (RoA) was measured for the 300nm sample. A 10 µl drop was placed in the middle of the sample and the stage was tilted at a speed of 90°/min. The tilting direction was parallel and perpendicular to the wrinkle's arrangement. When the drop displacement was parallel to the wrinkle's arrangement the RoA was ~55°, when perpendicular, the RoA was > 75°. This anisotropy effect suggests that the wrinkles hinder the mobility of the droplet when it displaces against the wrinkle's orientation.

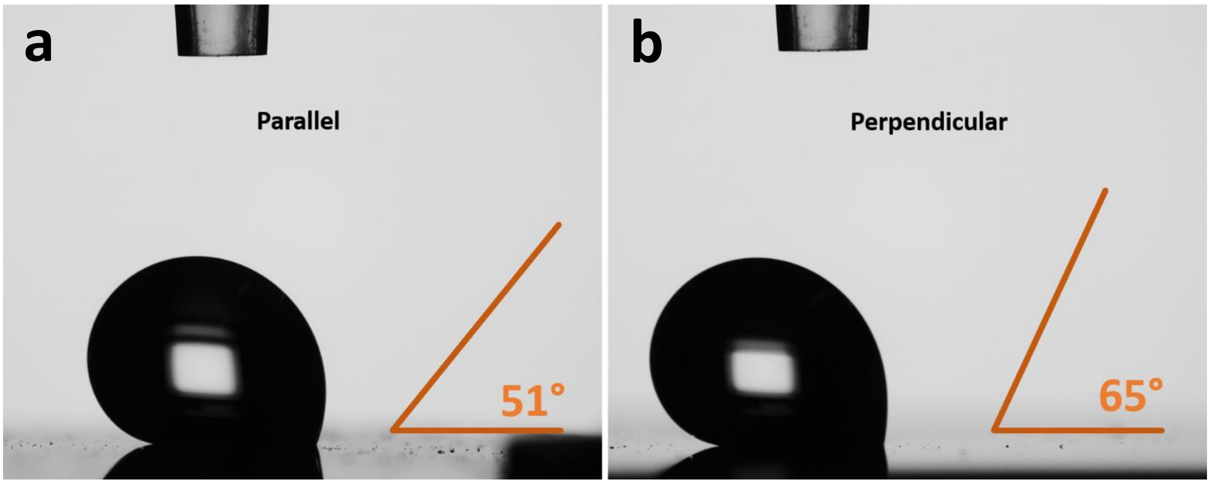

Figure S4- Photo of the droplet profile when the substrate was tilted parallelly (a) and perpendicularly (b) to the wrinkle direction. The angle indicated is the roll-off-angle, which evidently changes depending on the tilting direction.

Table S2- Values found from the fitting for each different volume of the droplets used, referred to the long wavelength

Volume (µl)	y_{int}	*st. dev.*	*b*	*st. dev.*	*adj. R*
3	2.4	0.3	-0.8	0.1	0.95
4	2.4	0.3	-0.8	0.1	0.92
5	2.4	0.2	-0.8	0.1	0.96
7	2.3	0.3	-0.8	0.1	0.94
10	2.4	0.2	-0.8	0.1	0.98
average	**2.4**	***0.3***	**-0.8**	***0.1***	**0.95**

Table S3- Values found from the fitting for each different volume of the droplets used, referred to the short wavelength

Volume (µl)	*yint*	*st. dev.*	*b*	*st. dev.*	*adj. R*
3	-1.0	0.2	0.7	0.1	0.93
4	-1.0	0.2	0.6	0.1	0.93
5	-0.9	0.2	0.6	0.1	0.95
7	-0.9	0.2	0.6	0.1	0.93
10	-0.9	0.2	0.6	0.1	0.96
average	**-0.9**	***0.2***	**0.6**	***0.1***	**0.94**

Section 4- Icing experiments

In *Figure S5* the surface of the 600 nm-coated sample is shown during the freezing experiment. Small water droplets tended to wedge in between the double peaks of a single wrinkle, while bigger droplets rolled off to the area defined by two wrinkles. It was observed that numerous dendrites would start growing from the droplet during freezing, leading to the formation of a continuous layer of ice over the surface. Once the temperature was raised, the ice melted, and the water droplets coalesced into

bigger drops positioned mainly in between wrinkles. The hydrophobic character was evident since the droplets did not form a continuous layer over the coating [36]. This was also suggesting the integrity of the coating itself.

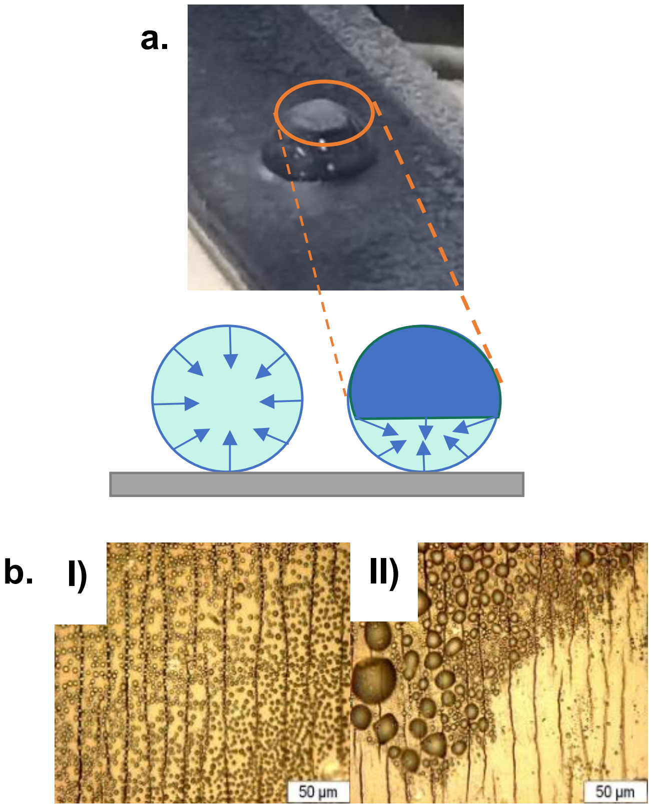

Figure S5- a. Image of a droplet deposited on the PDMS coated sample during freezing. It is possible to notice two phases, a white part on top of the freezing droplet is believed to be the ice formed. Freezing started from the outer surface of the droplet towards the inner body, as explained by the arrows. In specific the first side starting freezing was the top, observing a delay at the bottom of the droplet. b. In the plot the freezing delay caused in water droplet is compared among the samples with ordered and random wrinkles. The thickness of the coating lays on the x axes versus the delay measured.

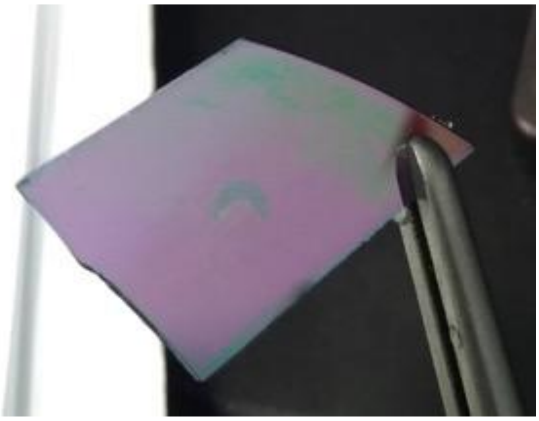

Figure S6- Image of the silicon wafer coated with pPFDA after the freezing experiment and the removal of the droplet. The coating appears damaged and loses its hydrophobic properties where the ice was formed.

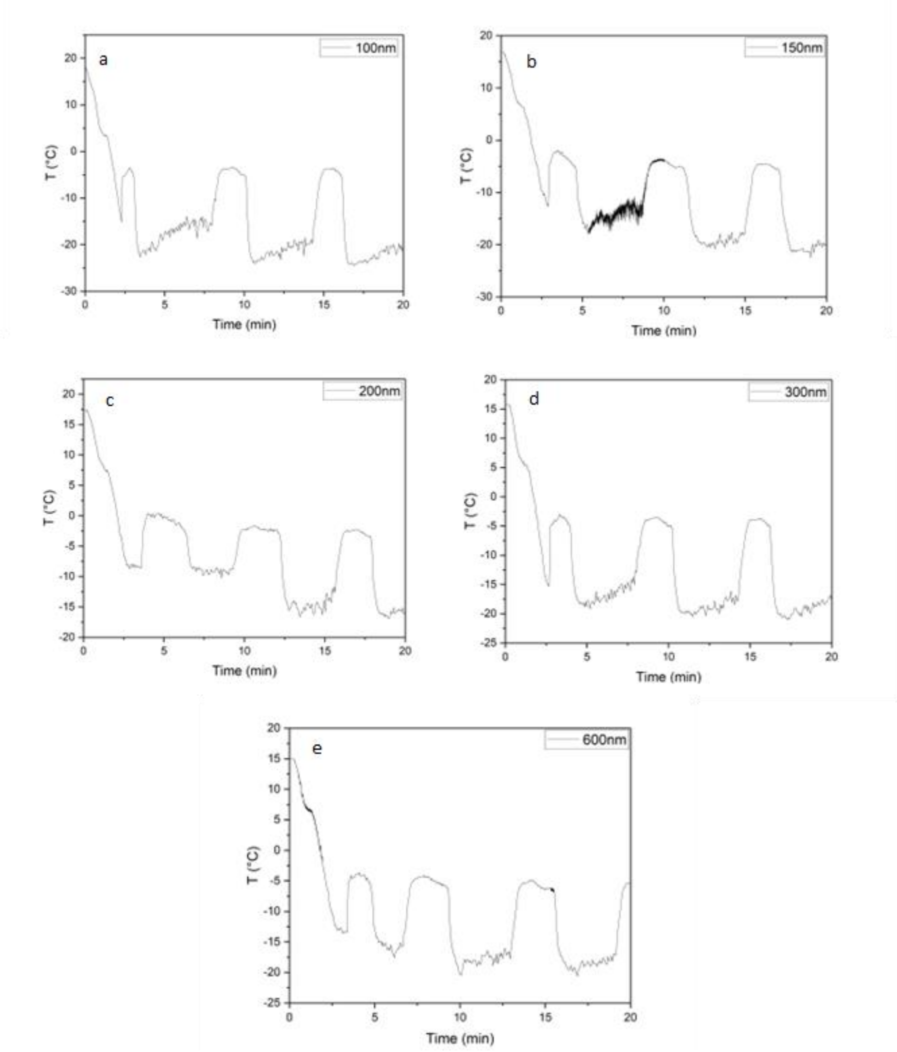

Figure S7- Temperature profiles of coated PDMS. Different thicknesses were investigated. A droplet of 10 µl was deposited and froze three times in a row at different cooling rates, respectively 20, 30, and 40 °C/min. Between each freezing event the substrate was heated up to 0 °C at a constant rate of 30 °C/min. a) 100 nm, b) 150 nm, c) 200 nm, d) 300 nm, e) 600 nm are the thicknesses of the samples.

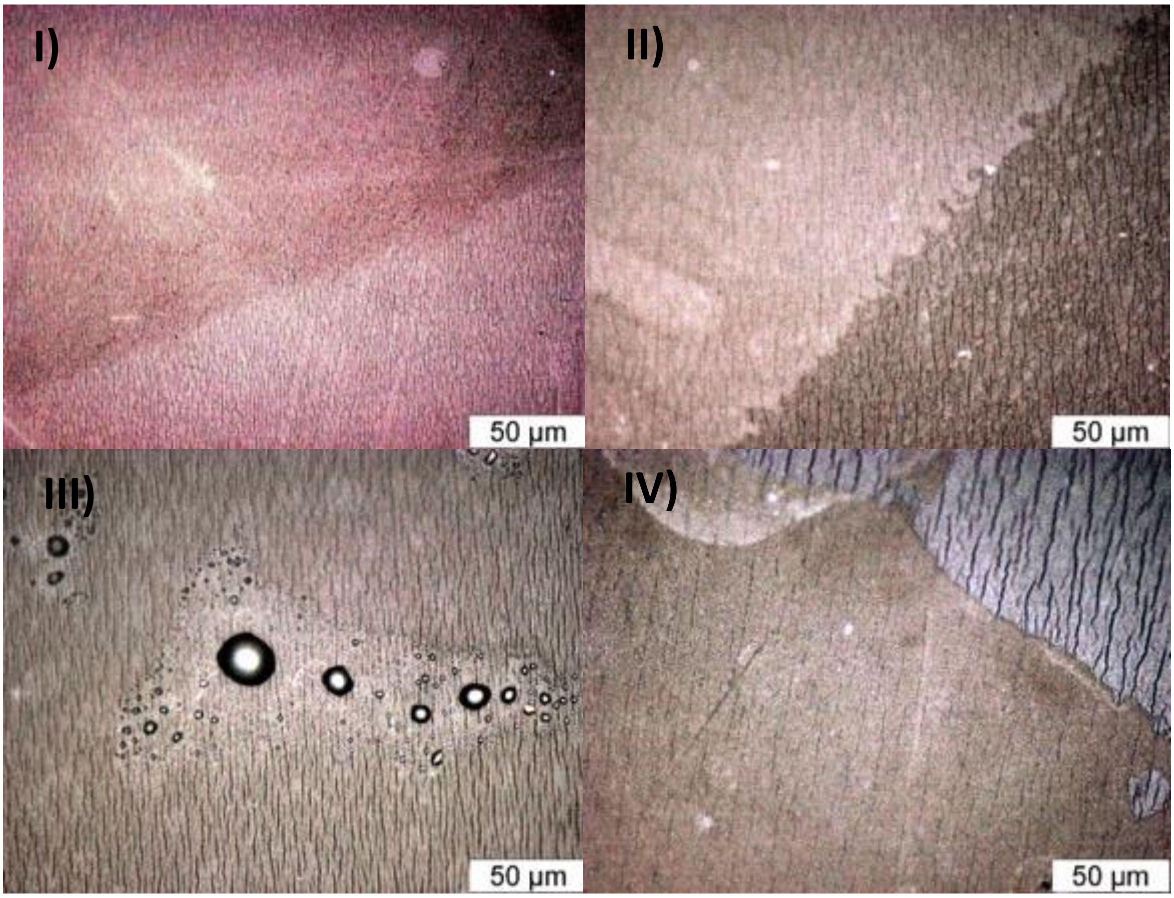

Figure S8- Optical Image of the ripped area for each sample tested I)100nm, II) 150nm, III) 200nm, IV) 300nm, 500x magnification

Table S4 – Measurement on the ripped areas on samples with profilometer

Original thickness of the coating (nm)	***Step (nm)***	***% removed coating***
100	30	31
150	29	20
200	63	32
300	110	37

References

1. B D Cassie, B. A. & Baxter, S. *OF POROUS SURFACES*.
2. Mondal, S., Phukan, M. & Ghatak, A. Estimation of solid-liquid interfacial tension using curved surface of a soft solid. *Proc. Natl. Acad. Sci. U. S. A.* **112**, 12563–12568 (2015).

3. Quéré, D. Wetting and roughness. *Annu. Rev. Mater. Res.* **38**, 71–99 (2008).
4. Zhang, X. *et al.* Flexible and mechanically robust superhydrophobic silicone surfaces with stable Cassie–Baxter state. *J. Mater. Chem. A* **4**, 14180–14186 (2016).
5. Rodríguez-Hernández, J. Wrinkled interfaces: Taking advantage of surface instabilities to pattern polymer surfaces. *Prog. Polym. Sci.* **42**, 1–41 (2015).
6. Scarratt, L. R. J., Hoatson, B. S., Wood, E. S., Hawkett, B. S. & Neto, C. Durable Superhydrophobic Surfaces via Spontaneous Wrinkling of Teflon AF. *ACS Appl. Mater. Interfaces* **8**, 6743–6750 (2016).
7. Koishi, T., Yasuoka, K., Fujikawa, S., Ebisuzaki, T. & Zeng, X. C. Coexistence and transition between Cassie and Wenzel state on pillared hydrophobic surface. *Proc. Natl. Acad. Sci.* **106**, 8435–8440 (2009).
8. Murakami, D., Jinnai, H. & Takahara, A. Wetting transition from the cassie-baxter state to the wenzel state on textured polymer surfaces. *Langmuir* **30**, 2061–2067 (2014).
9. Aït-Mokhtar, A., Amiri, O., Dumargue, P. & Bouguerra, A. On the applicability of Washburn law: study of mercury and water flow properties in cement-based materials. *Mater. Struct.* **37**, 107–113 (2004).
10. Li, K., Zhang, D., Bian, H., Meng, C. & Yang, Y. Criteria for Applying the Lucas-Washburn Law. *Sci. Rep.* **5**, 14085 (2015).
11. Phys. Rev. Lett. 99, 156001 (2007) - Spontaneous Breakdown of Superhydrophobicity. https://journals.aps.org/prl/abstract/10.1103/PhysRevLett.99.156001.
